# LATINOS’ PERCEPTIONS AND CONCERNS ABOUT ALZHEIMER’S DISEASE

**DOI:** 10.1093/geroni/igz038.1996

**Published:** 2019-11-08

**Authors:** Laura Y Cabrera

**Affiliations:** Michigan State University, East Lansing, Michigan, United States

## Abstract

Several studies indicate that Latinos are at higher risk of developing Alzheimer Disease (AD). While research has centered on African-American/White or Latino/non-Latino differences, there exists heterogeneity within those groups. Clustering Latinos under a single group in AD resources, neglects cultural, biological and environmental differences. To address this complexity we examine perceptions and concerns about AD symptoms, diagnosis, and care among Mexicans and Puerto Ricans via six focus groups. A priori variables for thematic exploration include familiarity, cultural beliefs, trust, privacy, notions of identity and personhood. We use a pragmatic neuroethics framework as a lens to discuss and assess our findings and related implications. This will help address the multidimensional and multidirectional nature of knowledge and communication about diagnosis, treatments and nature of AD. These findings will help to identify differences and similarities among two distinct Latino groups, thereby contributing to scholarship in the fields of Latino’s health, aging, and neuroethics.

